# Epidemiological Study of Uveal Melanoma from US Surveillance, Epidemiology, and End Results Program (2010–2015)

**DOI:** 10.1155/2020/3614039

**Published:** 2020-02-19

**Authors:** Yufeng Xu, Lixia Lou, Yijie Wang, Qi Miao, Kai Jin, Menglu Chen, Juan Ye

**Affiliations:** Department of Ophthalmology, The Second Affiliated Hospital of Zhejiang University, College of Medicine, 88 Jiefang Road, Hangzhou, Zhejiang 310009, China

## Abstract

**Purpose:**

Uveal melanoma is the most common intraocular malignancy, and the American Joint Committee on Cancer (AJCC) changed its staging methodology from 2010, incorporating notable changes into the T-staging. There were few literatures evaluating the epidemiological trend and risk factors of survival in multicenter longitudinal studies regarding the new staging system.

**Methods:**

We performed population-based cohort analyses using the Surveillance, Epidemiology, and End Results (SEER) database to identify patients with primary uveal melanoma from 2010 to 2015. Patients and potential prognosis indicators were extracted from SEER 18. Incidence rates, incidence rates ratios (IRR), annual percent changes (APC) in rate, hazard ratios (HR), 5-year accumulative overall survival (OS), and disease-specific survival (DSS) were calculated.

**Results:**

A total of 2631 patients for incidence analysis and 1142 patients for survival analysis were retrieved. The overall incidence of uveal melanoma was 4.637 per million (95% confidence interval (CI), 4.458–4.821), which was significantly elevated by average APC of 4.215% (*p* = 0.03). Females had significantly lower incidence (4.076 per million, IRR, 0.768, 95% CI, 0.710–0.832) with noticeable differences among age, race, origin, and laterality in sex-stratified analyses as well. Survival analyses revealed 5-year accumulative OS and DSS for patients with uveal melanoma of 61.8% and 66.5%, respectively. Age, AJCC stage, and radiation therapy were found to be consistent predictors in both univariate and multivariate analysis models.

**Conclusion:**

Incidence of uveal melanoma increased by significant APC and varied between genders. Determinants of survival included age at diagnosis, AJCC stage, and radiation therapy.

## 1. Introduction

Uveal melanoma is the most common primary intraocular malignancy, which most commonly arises from choroidal melanocytes (85–90%) [1–6]. Blurred vision (37.8%) is the most common symptom; however, as many as one-third of patients are asymptomatic at diagnosis [6]. Uveal melanoma and cutaneous melanoma act quite differently in etiopathogenesis and biological behaviors [7, 8]. So far, several risk factors, including light eyes, Caucasian population, and certain skin conditions such as cutaneous nevi [9–14], dysplastic nevus syndrome [15–17], iris nevi [14, 18–20], and *BAP1* mutation, have been identified [21–23]. On the basis of clinical examination alone, the diagnostic rate could reach around 99.5%, according to data from Collaborative Ocular Melanoma Study (COMS) [24]. Despite easy diagnosis, metastatic disease will be observed on about half of patients of metastatic diagnosis with 6–12 months' survival [2]. The management of localized melanoma can be divided into globe-preserving therapy and enucleation. The COMS trial showed there is no difference between ^125^I brachytherapy and enucleation against medium-sized choroidal melanomas in 15 years of follow-up [25].

Uveal melanoma was commonly classified into three sizes: small, medium, and large, historically [26–28]. However, since 2010, the American Joint Committee on Cancer (AJCC) has changed the staging system for uveal melanoma from AJCC sixth to the seventh edition, which incorporated notable changes into the T-staging (i.e., changes to the size criteria for T1–T4, ciliary body involvement, and amount of episcleral extension) [29, 30]. There were few literatures comparing the outcomes of new classification methodology with past results. We consider that it is a high priority to update the epidemiological trends in uveal melanoma and evaluate prognostic predictors of patient survival regarding the new staging system. The Surveillance, Epidemiology, and End Results (SEER) Program of the National Cancer Institute (NCI) is an important source of the longitudinal epidemiological study from multicenter population-based registries, which is updated annually and is available free of charge to the public. In this study, we used data from SEER 18 to analyze recent epidemiological trends of uveal melanoma, disease characteristics, and various potential predictors implicating patient survival.

## 2. Methods

A population-based longitudinal analysis for patients with a diagnosis of primary uveal melanoma was performed using the NCI SEER 18 database (http://www.seer.cancer.gov) via the SEER∗Stat software (version 8.3.5) in client server mode. This cancer registry captures 18 distinct population groups in 198 counties in the United States, which covers a 28% of the US population, including 23% of African Americans and 40% of Hispanics. It collects patients' data such as demographic information, cancer characteristics, initial treatment, and follow-up. Internal review board permission was not required because the database compiles publicly available information without personal identifiers.

We identified cases as primary uveal melanoma according to the *International Classification of Disease-Oncology*, *third edition* (ICD-O-3), morphology code *8720*–*8790* and site code *C69*.*3-C69*.*4*. The inclusion criteria of survival analysis were cases need to be microscopically confirmed during 2010–2015, active follow-up, and survival of not less than 2 months after diagnosis. We excluded the cases that were only confirmed via autopsy after death. Variables such as year of diagnosis, age at diagnosis, sex, race (White, Black, American Indian/Alaska Native (AIAN), and Asian or Pacific Islander (API)), origin (non-Hispanic and Hispanic), primary laterality, marital status, summary stage, AJCC stage, months of survival, surgery, metastasis at diagnosis, radiation treatment, chemotherapy, and cause-specific classification of death were extracted.

Age-adjusted incidence rates (cases per million person-years, using 2000 US Standard Population as reference population), incidence rate ratios (IRR), and annual percent changes (APC) were calculated via the SEER∗Stat software. Age at diagnosis, race, origin, and primary laterality were taken into account among sex-stratified analyses.

The optimal cutoff values for age range were determined using the X-tile software (http://www.tissuearray.org/rimmlab) in survival analyses. Survival status, survival time, and age of patients were loaded as parameters of “Censor,” “Survival Time,” and “Marker” in the X-tile software, in order to discover the difference between age ranges. The overall survival (OS) and disease-specific survival (DSS) were plotted by the Kaplan–Meier method, with differences tested by log-rank algorithm. Multivariate Cox proportional hazards regression model was adopted to assess the predictive performance of covariates. Statistical analyses and graphics were performed using IBM SPSS statistics, version 24.0 (SPSS, Inc, Chicago, IL); *p* value less than 0.05 was considered to be statistically significant unless otherwise specified.

## 3. Results

From the SEER 18 database between 2010 and 2015, a total of 2631 cases were pooled for incidence analyses. Further, after filtered by inclusion criteria mentioned above, 1142 cases were extracted for survival analyses. In incidence analysis, patient age ranging from 60 to 80 years (49.0%), White race (96.3%), and non-Hispanics (94.3%) counted for the majority proportion of uveal melanoma patients. Overall incidence of uveal melanoma was 4.637 per million (95% confidence interval (CI), 4.458–4.821), with a significantly lower IRR of 0.768 (95% CI, 0.710–0.832) in female. Aging population (IRR, age 60–80, 7.060, 95% CI, 6.498–7.669; age >80, 6.194, 95% CI, 5.351–7.146), White race (IRR, Black, 0.076, 95% CI, 0.048–0.114; AIAN, 0.164, 95% CI, 0.061–0.350; API, 0.098, 95% CI, 0.066–0.139), and non-Hispanics (IRR, Hispanics, 0.350, 95% CI, 0.291–0.416) had considerable higher incidence ([Supplementary-material supplementary-material-1], Table 1). Given the significant gender variation, we conducted sex-stratified subgroup analyses including age range, race, origin, and primary laterality. Similar incidence patterns remained (Table 1).

Investigation of temporal patterns in overall uveal melanoma incidence from SEER 18 revealed a significant increasing trend (APC, 4.215%, *p*=0.03) during 2010–2015. Further examination of sex-stratified subgroup analyses unveiled upward trends in female ranging from 60 to 80 (APC, 5.877%, *p*=0.044), White males (APC, 4.515%, *p*=0.043), and non-Hispanics (APC, male, 4.431%, *p*=0.039; female, 4.898%, *p*=0.035) (Table 2).

A total of 1142 cases were selected for survival analyses, among which 44.8% were females and 55.2% were males. The mean age at diagnosis was 61.5 years. Surgical treatment was carried out for 42.7% of patients, and 60.5% were treated with different kinds of radiation therapy. The majority of cases were classified in AJCC stage II (38.3%) and the rest distributed in stage I (15.1%), stage II (19.4%), and stage IV (2.4) with 24.9% unknown stage. Other tumor characteristics about race, origin, primary laterality, marital status, summary stage, metastasis, and chemotherapy were presented in [Supplementary-material supplementary-material-1].

**Figure 1:**
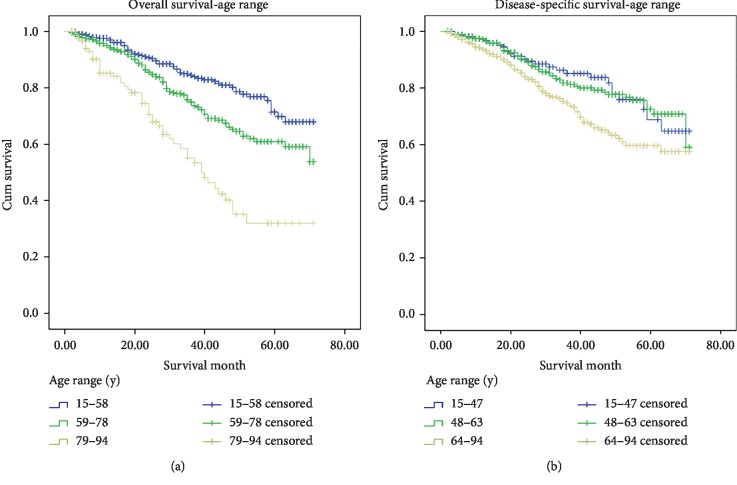
Survival analysis of patients with choroidal melanoma using the Kaplan–Meier analysis. (a) Kaplan–Meier estimates of overall survival for all cases by age range and (b) disease-specific survival for all cases by age range.

Survival curves from Kaplan–Meier (univariate analyses) (Figure 1) revealed that the 5-year accumulative OS and DSS for uveal melanoma was 61.8% and 66.5%, respectively (Table 3). Both OS and DSS showed significant higher survival rates in young patients (age range was determined by the X-tile software, Figure S in Supplementary file). (OS: group a, 15–58; group b, 59–78; group c, 79–94. DSS: group A, 15–47; group B, 48–63; group C, 63–94.) (OS: group b, 61.0%, *p*=0.001, group c, 31.9%, *p* < 0.001; DSS: group C, 59.7%, *p*=0.01). Besides, White race, higher AJCC stage, severe summary stage, distant metastasis, and no radiation treatment displayed considerably lower survival rates in OS and DSS analyses. Unexpectedly, we noticed significantly worse prognostic outcome in surgically treated patients (OS, 52.6%, *p* < 0.001; DSS, 55.3%, *p* < 0.001).

We used the multivariate analysis model (COX regression) to ascertain the independent effects of case variables (Table 4). In the OS analysis, people in group b, 59–78, and group c, 79–94, (HR, 1.532, 95% CI, 1.122–2.093, *p* = 0.007; HR, 3.670, 95% CI, 2.500–5.389, respectively) and higher AJCC stage (stage II, HR, 5.098, 95% CI, 2.204–11.791, *p* < 0.001; stage III, HR, 4.347, 95% CI, 1.738–10.876, *p* = 0.003) showed consistent prognosis. While in the DSS analysis, besides the abovementioned two risk factors, radiation therapy seemed to be effective to prolong patient survival (HR, 0.551, 95% CI, 0.329–0.925, *p* = 0.024).

## 4. Discussion

Several published studies reported the mean age-adjusted incidence of uveal melanoma at different time periods in the United States: 4.3 per million from 1973 to 1997 by Singh et al.; 5.1 per million from 1973 to 2008 by Singh et al.; and 5.2 per million from 1973 to 2013 by Aronow et al. [1, 31, 32]. Overall age-adjusted incidence remained stable over the past 4 decades with significant higher incidence in male subjects, claimed by the abovementioned studies, and some other studies demonstrated the disease had no sex preference [15]. The median age of diagnosis is around 60 years; however, the peak range seemed to be between 70 and 79 years [4, 6, 33]. In our current study, we identified an overall incidence of uveal melanoma of 4.637 per million (95% CI, 4.458–4.821), which is similar to earlier reports, and confirmed the sexual variation of incidence. Elderly people, White race, and non-Hispanic population tended to be more vulnerable to have uveal melanoma. However, we did notice a considerable overall APC of 4.125% (*p* = 0.03) during 2010–2015. Meanwhile, the increasing trends existed in mid-aged (60–80 years) people, female elderly people, White males, and non-Hispanic population. Unlike retinoblastoma, there are few studies that focus on laterality of uveal melanoma. Also, sex-stratified analysis of uveal melanoma laterality is scarce as well. It is quite understandable that males had a significantly higher incidence of uveal melanoma in both eyes than females, considering the men's higher total age-adjusted incidence. However, our relatively short follow-up duration, higher *p* value set-up (0.05), and marginally statistical significance cannot be neglected. Though a higher IRR of a male left eye (1.141 (1.023–1.272)) and total right eye APC (4.389%, *p* = 0.046) might be true reflection of reality, they might also be false positive as well. Hence, the data should be interpreted with caution, and further studies are needed to test the conclusion.

In terms of potential prognostic predictors, it has been implicated that older age at diagnosis and male gender correlate with reduced survival [34], which is consistent with the findings in UK [35], Sweden [36], and Denmark [37]. Though no sex differences were found in the COMS, maximum basal tumor diameter together with age was the strongest predictor of mortality for uveal melanoma. [38] Yet researchers are not able to determine to what extent these associations are results of bias generating from confounding factors [39]. In our study, we adopted the X-tile software to optimize the age range cutoff instead of subjective classification. According to the results of X-tile, we divided patients into 3 age groups of OS and DSS analyses (due to different causes of mortality): group a, 15–58; group b, 59–78; and group c, 79–94 for OS and group A, 15–47; group B, 48–63; and group C, 63–94 for DSS. It is our attempt to quantize the age risk, and interpret it with caution. More importantly, the staging system of uveal melanoma witnessed critical changes when the AJCC 7th version was started to be practiced from 2010. It categorizes tumor based on their size, including tumor basal diameter and height, also taking into account ciliary body involvement and episcleral extension. Uveal melanoma survival decreases rapidly with increasing stage. Estimates of death at 5 years are 4% for T1, 8% for T2, 19% for T3, and 30% for T4 lesions, respectively. [40] We identified that 5-year accumulative OS and DSS for uveal melanoma was 61.8% and 66.5%, respectively. Compared with previous studies, the survival period seemed to be lower, which may be due to short recruiting time window. In our univariate analyses, older age, White race, higher AJCC stage, severe summary stage, distant metastasis, and no radiation treatment showed significantly lower survival rates in OS and DSS analyses. However, only factors of age, AJCC stage, and radiation persisted to demonstrate prediction potential in multivariate analyses. Despite our restriction of observation time period, this study might act as a supplement to other long-time follow-up literatures using the old staging system.

We observed reduced survival period in patients who underwent primary-site surgery. Though it lost statistical significance in multivariate analyses, it still brought up the controversial topic of treatment decision-making. Our analyses indicated that patients who underwent radiation therapy showed improved survival period, which supported the COMS trial comparing radiation with enucleation during 15 years of follow-up [25]. There has been a significant shift from local resection and enucleation toward radiotherapy in the United States [41]. Radiation therapy displayed wonderful local control and globe preservation, but long-term vision loss is inevitable. Some alternative treatments like transpupillary thermal therapy also showed efficacy in residual uveal melanoma [42, 43]. Moreover, a series of novel approaches are currently developing. For example, human tissue factor VII is commonly overexpressed in uveal melanoma and contributes to tumor growth, thrombosis, angiogenesis, and metastasis [44]. ICON-1, which is a synthetic structural variant of factor VII, binds to tumor cells and initiates a signal cascade targeting immune cells to pathological tissue (NCT02771340). However, we still wait for long-term outcomes from different research studies.

While SEER registries give us the easy access to large-scale population-based data from multicenters, which is very helpful to do longitudinal analysis on tumor epidemiological study, there are some inherent limitations as well. The database lacks information such as detail surgical depiction, comorbidities, hospital volume, and tumor recurrence. There are also concerns regarding misclassification among different registries. In terms of statistical methodology, under a certain sample scale, a relatively higher *p* value cutoff (0.05) might cause false significance, which could give chances of deception and misinterpretation of data. Though we have compared similar studies using the SEER database with different case numbers and follow-up duration, most of which chose 0.05 as *p* set-up. [1, 32, 33, 45–47], we should still be prudent when applying these results.

## 5. Conclusion

In summary, we identified the incidence of 4.637 per million population of uveal melanoma during 2010–2015. There is a significantly increasing APC of 4.215% and continued gender preference toward incidence. Age at diagnosis, AJCC stage, and radiation therapy may be potential predictors of prognosis. These findings may raise public attention to monitor epidemiological trends, prognostic factors, and treatment selection of uveal melanoma. Our study might supplement previous long-time follow-up literatures using the old staging system. Further studies are needed to confirm our results.

## Figures and Tables

**Table 1:** Sex-stratified uveal melanoma age-adjusted incidence rates and IRRs from the SEER 18 registries research database, 2010–2015 (2631 cases included).

Characteristic	Male			Female			Female-male IRR (95% CI)	Overall IRR (95% CI)
	No. of patients^a^	Incidence rate^b^	IRR (95% CI)	No. of patients	Incidence rate^b^	IRR (95% CI)		
Total	1405	5.306	Reference	1226	4.076	**0.768 (0.710–0.832)**	**0.768 (0.710–0.832)**	**0.768 (0.710–0.832)**
Age range								
<60	603	2.573	Reference	506	2.129	Reference	**0.827 (0.732–0.935)**	Reference
60–80	689	19.148	**7.441 (6.643–8.336)**	599	14.366	**6.749 (5.970–7.629)**	**0.750 (0.670–0.840)**	**7.060 (6.498–7.669)**
>80	113	18.973	**7.373 (5.973–9.033)**	121	11.825	**5.555 (4.506–6.807)**	**0.623 (0.478–0.814)**	**6.194 (5.351–7.146)**
Race								
White	1348	6.415	Reference	1185	5.114	Reference	**0.797 (0.735–0.864)**	Reference
Black	14	0.535	**0.083 (0.042–0.146)**	11	0.360	**0.070 (0.034–0.127)**	0.673 (0.267–1.687)	**0.076 (0.048–0.114)**
AIAN	2	0.456	**0.071 (0.009–0.298)**	5	1.354	**0.265 (0.079–0.636)**	2.968 (0.405–31.448)	**0.164 (0.061–0.350)**
API	18	0.701	**0.109 (0.064–0.175)**	14	0.443	**0.087 (0.047–0.148)**	0.632 (0.286–1.374)	**0.098 (0.066–0.139)**
Origin								
Non-Hispanic	1347	5.969	Reference	1137	4.424	Reference	**0.741 (0.683–0.805)**	Reference
Hispanic	58	1.596	**0.267 (0.197–0.353)**	89	2.008	**0.454 (0.359–0.567)**	1.258 (0.879–1.823)	**0.350 (0.291–0.416)**
Primary laterality								
Right	658	0.247	Reference	625	0.208	Reference	**0.839 (0.749–0.940)**	Reference
Left	745	0.282	**1.141 (1.023–1.272)**	595	0.198	0.953 (0.848–1.071)	**0.701 (0.627–0.784)**	1.045 (0.966–1.131)

Abbreviation: SEER, Surveillance, Epidemiology, and End Results; IRR, incidence rates ratios; AIAN, American Indian/Alaska Native; API, Asian or Pacific Islander. ^a^Total amount may not be 2631 due to exclusion of cases with unknown information. ^b^Incidence rates are based on the number of persons diagnosed as having uveal melanoma per 1,000,000 person-years, age adjusted using the 2000 US population standard. Bold letter indicates that measurements are statistically significant compared with references (*p* < 0.05).

**Table 2:** Sex-stratified trends in uveal melanoma from the SEER 18 registries research database, 2010–2015 (2631 cased included).

Characteristic^a^	Male		Female		Overall	
	Rate/trend, %	*p* value	Rate/trend, %	*p* value	Rate/trend, %	*p* value
Total	5.140	0.056	−0.703	0.721	4.215	**0.03** ^*∗*^
Age range						
<60	2.028	0.230	1.307	0.707	1.554	0.272
60–80	2.368	0.271	5.877	**0.044** ^*∗*^	4.134	0.057
>80	6.238	0.152	0.655	0.913	3.434	0.344
Race						
White	4.515	**0.043** ^*∗*^	4.973	0.051	4.733	**0.030** ^*∗*^
Black	1.587	0.909	−4.330	0.790	−1.269	0.930
AIAN	N/A^b^	N/A^b^	N/A^b^	N/A^b^	N/A^b^	N/A^b^
API	−2.411	0.881	−15.039	0.478	−9.126	0.550
Origin						
Non-Hispanic	4.431	**0.039** ^*∗*^	4.898	**0.035** ^*∗*^	4.646	**0.022** ^*∗*^
Hispanic	−2.058	0.552	2.509	0.617	0.847	0.698
Primary laterality						
Right	3.368	0.167	5.741	0.269	4.389	**0.046** ^*∗*^
Left	4.305	0.102	3.675	0.314	3.966	0.132

Abbreviation: SEER, Surveillance, Epidemiology, and End Results; IRR, incidence rates ratios; AIAN, American Indian/Alaska native; API, Asian or Pacific Islander. ^a^Total amount may not be 2361 due to exclusion of cases with unknown information. ^b^Values were smaller in subgroup and hence unavailable. Bold letter indicates that measurements are statistically significant compared with references (*p* < 0.05). ^*∗*^*p* < 0.05.

**Table 3:** Univariate analysis of overall and disease-specific survival (1142 cases included).

Characteristic	*p* value for log rank		Cumulative survival rate at 5 y, %	
	OS	DSS	OS	DSS
Overall			61.8	66.5
Age range^a^				
Young	Reference	Reference	71.4	68.9
Mid	**0.001** ^*∗∗*^	0.798	61.0	72.6
Elder	**<0.001** ^*∗∗∗*^	**0.01** ^*∗*^	31.9	59.7
Sex				
Female	Reference	Reference	62.9	68.0
Male	0.166	0.379	60.8	65.2
Race				
White	Reference	Reference	60.2	65.5
Others	**0.042** ^*∗*^	**0.049** ^*∗*^	84.4	91.1
Origin				
Hispanic	Reference	Reference	57.6	66.7
Non-Hispanic	0.212	0.143	61.2	61.4
Laterality				
Right	Reference	Reference	60.2	67.9
Left	0.698	0.475	61.7	65.2
AJCC stage				
I	Reference	Reference	88.0	93.6
II	**<0.001** ^*∗∗∗*^	**<0.001** ^*∗∗∗*^	67.4	72.9
III	**<0.001** ^*∗∗∗*^	**<0.001** ^*∗∗∗*^	46.3	49.5
IV	**<0.001** ^*∗∗∗*^	**<0.001** ^*∗∗∗*^	13.7^b^	14.4 ^b^
Summary stage				
Localized	Reference	Reference	64.3	69.3
Regional	**0.013** ^*∗*^	**0.02** ^*∗*^	60.1	63.4
Distant	**<0.001** ^*∗∗∗*^	**<0.001** ^*∗∗∗*^	13.5^b^	11.2 ^b^
Metastasis at diagnosis				
None	Reference	Reference	62.6	67.4
Distant	**<0.001** ^*∗∗∗*^	**< 0.001** ^*∗∗∗*^	13.5^b^	11.2 ^b^
Surgery				
Performed	Reference	Reference	54.7	59.5
Not performed	**<0.001** ^*∗∗∗*^	**<0.001** ^*∗∗∗*^	67.3	71.8
Radiation				
Performed	Reference	Reference	69.0	74.2
Not performed	**<0.001** ^*∗∗∗*^	**<0.001** ^*∗∗∗*^	52.6	55.3
Chemotherapy				
Performed	Reference	Reference	56.0	56.0
Not performed	0.160	0.059	61.3	66.8
Marital status				
Married	Reference	Reference	62.1	66.1
Others	0.824	0.61	61.4	67.1

Abbreviations: OS, overall survival; DSS, disease-specific survival; AJCC, American Joint Committee on Cancer. ^a^Age stratification was different in OS and DSS, according to results of X-tile process. ^b^Due to lack of patient sample, the longest follow-up time was 46 months. We used the latest survival data instead of accurate 5-year accumulative survival rate. Bold letter indicates that measurements are statistically significant compared with references (*p* < 0.05) ^*∗*^*p* < 0.05; ^*∗∗*^*p* < 0.01; ^*∗∗∗*^*p* < 0.001.

**Table 4:** Cox proportional hazard ratio (HR) for overall and disease-specific survival (1142 cases included).

Characteristic	Overall survival		Disease-specific survival	
	HR (95% CI)	*p* value	HR (95% CI)	*p* value
Age range^a^		<0.001^*∗∗∗*^		0.001^*∗∗∗*^
Young	Reference		Reference	
Mid	1.532 (1.122–2.093)	**0.007** ^*∗∗*^	1.005 (0.627–1.608)	0.985
Elder	3.670 (2.500–5.389)	**<0.001** ^*∗∗∗*^	1.721 (1.107–2.674)	**0.016** ^*∗*^
Race		0.133		0.09
Others	Reference		Reference	
White	2.151 (0.791–5.851)	0.133	2.715 (0.867–8.598)	0.090
AJCC stage		**0.002** ^*∗∗*^		**0.002** ^*∗∗*^
I	Reference		Reference	
II	5.098 (2.204–11.791)	**<0.001** ^*∗∗∗*^	9.174 (3.103)	**<0.001** ^*∗∗∗*^
III	4.347 (1.738–10.876)	**0.003** ^*∗∗*^	8.337 (2.617–26.553)	**<0.001** ^*∗∗∗*^
IV	N/A^b^	N/A^b^	N/A^b^	N/A^b^
Summary stage		0.999		0.985
Localized	Reference		Reference	
Regional	0.969 (0.505–1.875)	0.924	1.074 (0.526–2.195)	0.845
Distant	0.974 (0.428–2.218)	0.950	1.173 (0.474–2.903)	0.730
Metastasis at diagnosis		0.613		0.49
None	Reference		Reference	
Distant	0.810 (0.358–1.834)	0.613	1.339 (0.584–3.071)	0.49
Surgery		0.829		0.548
Not performed	Reference		Reference	
Performed	0.890 (0.554–1.429)	0.63	0.749 (0.443–1.268)	0.282
Radiation		0.080		**0.024** ^*∗*^
Not performed	Reference		Reference	
Performed	0.657 (0.410–0.1.052)	0.080	0.551 (0.329–0.925)	**0.024** ^*∗*^
Chemotherapy		0.204		0.520
Not performed	Reference		Reference	
Performed	0.611 (0.285–1.307)	0.204	0.779 (0.364–1.667)	0.520

Abbreviations: AJCC, American Joint Committee on Cancer. ^a^Age stratification was different in OS and DSS, according to results of X-tile process. ^b^Values were smaller in subgroup and hence unavailable. Bold letter indicates that measurements are statistically significant compared with references (*p* < 0.05). ^*∗*^*p* < 0.05; ^*∗∗*^*p* < 0.01; ^*∗∗∗*^*p* < 0.001.

## Data Availability

The raw data about uveal melanoma cases supporting this research are from http://www.seer.cancer.gov. The proceeded data are all available from the corresponding author upon request (Yejuan@zju.edu.cn).

## Supplementary Materials

Supplementary Materials Figure S: X-tile analysis of survival based on age range. (A) Overall survival; (B) disease-specific survival. Table S1. Uveal melanoma age-adjusted incidence rates and IRRs from the SEER 18 registries research database, 2010–2015 (2631 cases included) Table S2: Demographics and tumor characteristics of 1142 patients from the SEER 18 registries research database, 2010–2015.
