# Supplementary material for: Epidemiological Study of Uveal Melanoma from US Surveillance, Epidemiology, and End Results Program (2010–2015)
Source: J Ophthalmol. 2020 Feb 19;2020:3614039. doi: 10.1155/2020/3614039 (PMC7049826; doi:10.1155/2020/3614039)
Supplement: Supplementary Materials — Figure S: X-tile analysis of survival based on age range. (A) Overall survival; (B) disease-specific survival. Table S1. Uveal melanoma age-adjusted incidence rates and IRRs from the SEER 18 registries research database, 2010–2015 (2631 cases included) Table S2: Demographics and tumor characteristics of 1142 patients from the SEER 18 registries research database, 2010–2015. [file 3614039.f1.pdf]

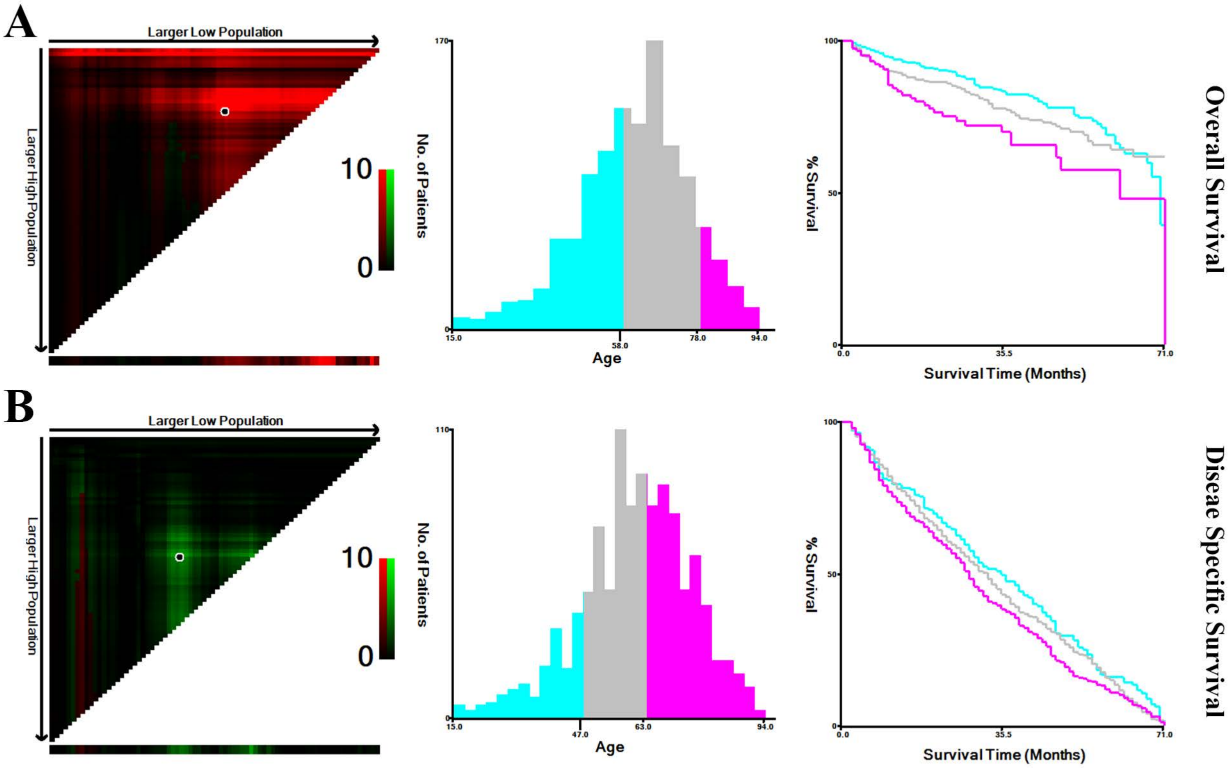

A
Larger Low Population
Larger High Population
10
0
170
No. of Patients
0
15.0
58.0
78.0
94.0
Age
100
% Survival
50
0
0.0
35.5
71.0
Survival Time (Months)
Overall Survival
B
Larger Low Population
Larger High Population
10
0
110
No. of Patients
0
15.0
47.0
63.0
94.0
Age
100
% Survival
50
0
0.0
35.5
71.0
Survival Time (Months)
Diseae Specific Survival

Table S1. Uveal melanoma Age-adjusted Incidence Rates and IRRs from the SEER 18 Registries Research Database, 2010–2015 (2631 cases included)

Characteristic	No of Patients[a]	Percentage[b], %	Incidence Rate (95% CI)	IRR (95% CI)
**Total**	2631	100	4.637 (4.458-4.821)	N/A
**Sex**				
Male	1405	53.4	5.306 (5.025-5.985)	Reference
Female	1226	46.6	4.076 (3.847-4.316)	**0.768 (0.710-0.832)**
**Age Range**				
< 60	1109	42.2	2.347 (2.209-2.492)	Reference
60-80	1288	49.0	16.569 (15.656-17.520)	**7.060 (6.498-7.669)**
> 80	234	8.9	14.537 (12.728-16.535)	**6.194 (5.351-7.146)**
**Race**				
White	2533	96.3	5.712 (5.487-5.944)	Reference
Black	25	1.0	0.435 (0.276-0.648)	**0.076 (0.048-0.114)**
AIAN	7	0.2	0.934 (0.350-1.998)	**0.164 (0.061-0.350)**
API	32	1.2	0.557 (0.378-0.793)	**0.098 (0.066-0.139)**
**Origin**				
Non-Hispanic	2482	94.3	5.135 (4.930-5.347)	Reference
Hispanic	147	5.6	1.795 (1.502-2.126)	**0.350 (0.291-0.416)**
**Primary Laterality**				
Right	1283	48.8	2.260 (2.136-2.390)	Reference
Left	1340	50.9	2.361 (2.234-2.494)	1.045 (0.966-1.131)

Abbreviation: SEER, Surveillance, Epidemiology, and End Results; IRR, Incidence rate ratio; AIAN, American Indian/Alaska Native; API, Asian or Pacific Islander;

[a] Total amount may not be 2631 due to removing cases with unknown information.

[b] Percentages may not total 100% because of rounding and removing cases with unknown information.

Bold letter indicates that measurements are statistically significant compared to references ($p < 0.05$).

Table S2. Demographics and Tumor Characteristics of 1,142 Patients from the SEER 18 Registries Research Database, 2010–2015

Characteristic	No. of Patients	Value[a]
**Sex, %**		
Female	512	44.8
Male	630	55.2
**Age, y**		
Mean (SD)		61.5 (14.3)
Median (range)		63 (15-94)
**Age Range, %**		
< 60	490	42.9
60-80	560	49.0
> 80	92	8.1
**Race, %**		
White	1091	95.5
Black	17	1.5
Others	21	1.8
Unknown	13	1.1
**Origin, %**		
Hispanic	66	5.8
Non-Hispanic	1076	94.2
**Primary Laterality, %**		
Right	553	48.4
Left	586	51.3
Pair	2	0.2
Unknown	1	0.1
**Marital Status, %**		
Single (Never Married)	185	16.2
Married	660	57.8
Divorced	85	7.4
Widowed	104	9.1
Separated	9	0.8
Unknown	99	8.7
**Summary Stage, %**		
Localized	968	84.8
Regional	72	6.3
Distant	26	2.3
Unknown	76	6.7
**AJCC Stage, %**		
I	173	15.1
II	437	38.3
III	221	19.4
IV	27	2.4
Unknown	284	24.9

**Metastasis at Diagnosis, %**		
None	1086	95.1
Distant	26	2.3
Unknown	30	2.6
**Surgery, %**		
Performed	488	42.7
Not Performed	654	57.3
**Radiation Subtype, %**		
Beam Radiation	197	17.3
Radioactive Implants	362	31.7
Radioisotopes	127	11.1
Beam with Implants or Isotopes	5	0.4
None/Unknown	451	47.4
**Chemotherapy, %**		
Performed	30	2.6
Not Performed	1112	97.4

Abbreviation: SEER, Surveillance, Epidemiology, and End Results; AJCC, American Joint Committee on Cancer.

[a] Percentages may not total 100% because of rounding.
